# Supplementary material for: Are carbon emissions trading and green financial instruments synergistic? -Comprehensive quantitative research based on content analysis
Source: PLoS One. 2024 Mar 7;19(3):e0298601. doi: 10.1371/journal.pone.0298601 (PMC10919601; doi:10.1371/journal.pone.0298601)
Supplement: S1 Data — (ZIP) [file pone.0298601.s001.zip › Data/地级市Z_F工作报告.zip!/║╙─╧/╫ñ┬φ╡Ω╩╨/└┤╫╘╣1⁄2╓┌║┼í╛┬φ┐╦╩2╛▌═°í┐_2019.docx]

各位代表：

现在，我代表县人民政府，向大会报告工作，请予审议，并请各位政协委员和其他列席同志提出意见。

一、2018年工作回顾

2018年，在市委、市政府和县委的领导下，在县人大及其常委会和县政协的监督支持下，全县上下坚持以习近平新时代中国特色社会主义思想为指导，认真贯彻党的十九大和十九届二中、三中全会精神，贯彻新发展理念，牢牢把握高质量发展要求，以供给侧结构性改革为主线，着力稳增长保态势、调结构抓转型、防风险促稳定，加快“四个泌阳”建设，高质量发展实现良好开局，圆满完成县人大十四届二次会议确定的各项目标任务。全年实现生产总值241.8亿元，增长9%；固定资产投资增长11.4%；社会消费品零售总额增长11.2%；规模以上工业增加值增长8.6%；财政一般公共预算收入11亿元，增长22.2%；一般公共预算支出57.9亿元，增长22.1%。主要经济指标位居全市前列。

（一）产业质效不断提高。工业经济平稳运行。产业集聚区功能更加完善，完成固定资产投资46.5亿元、增长8.1%，规上工业主营业务收入297.5亿元、增长16.8%，增加值69亿元、增长16%。夏南牛、食用菌、电子电器产业集群发展壮大，夏南牛产业园内肉牛屠宰生产线、牛肉熟食加工、牛血生物制剂和冷链产品建设等五个亿元以上项目先后建成投产，夏南牛科技公司全面建成。投资16.5亿元的鲜菇坊食用菌高科技产业园、投资9亿元的嘉沁食用菌工厂化循环种植与生产加工项目正在建设，中国食用菌院士工作站挂牌设立，并成功举办食用菌产业高峰论坛，食用菌产值实现50亿元，出口创汇突破1亿美元。电子产业园内东吉电子、吉奥电子、佳源电子等项目有序推进。60个省、市重点项目完成投资395.43亿元，占年度投资计划的140.7%。现代农业稳步发展。粮食总产达84.13万吨，持续实现稳产增产。完成粮改饲试点项目6万亩，通过国家农业农村部年度绩效考评。夏南牛、食用菌、烟叶等特色农业优势凸显，夏南牛存栏达到36万头，我县被国家农业农村部等九部门评为“泌阳夏南牛中国特色农产品优势区”。食用菌总产量达到38万吨，亿健食用菌农业产业化联合体被认定为省级农业产业化联合体。烟叶产量8.53万担，实现烟叶税2725万元。全县新型农业经营主体达到2100家，新增雨露菌业、泌花食品2家省级农业产业化龙头企业和6家市级农业产业化龙头企业，市级以上农业产业化龙头企业增加到39家。全面完成农村土地确权颁证工作，提前完成“两区”划定，农村集体资产清产核资稳步推进。有效防控非洲猪瘟疫情。现代服务业加快发展。第三产业实现增加值95.9亿元，增长12.3%。加强商务中心区建设，碧桂园城市综合体、上海洲业国际汽车城、中州国际商城二期项目全面启动。现代物流园区初具规模，投资15亿元汇融物流和投资3亿元新邦物流基本建成投产。实现电商交易额35亿元、网络零售额10.1亿元。房地产业健康发展，投资增长18.3%，销售商品房45.14万平方米。金融业持续稳定发展，新增贷款11.44亿元、同比增长15%，31家企业集中在中原股权交易中心挂牌，完成4起非法集资案件处置，县农商行正式挂牌开业。旅游业持续发展，接待游客386.5万人次，实现旅游综合收入3.49亿元。

（二）城镇形象明显提升。新型城镇化进程不断加快，城镇化率提高1.5个百分点、达到41.56%。城市建设和管理连续六年位居全市前列。规划设计得到加强。编制完成《泌阳县城市总体规划》实施评估等6个专项规划及13个片区控制性详细规划，完成商务中心区城市设计规划编制。完成乡镇总体规划修编和48个行政村的实用性村庄规划，建成19个乡镇规划展览室。城区功能不断完善。融资20亿元，实施47项重点城建工程。新建改建花园路、盘古大道、北二环路、棠西路等道路15条，打通行政路、车站路中段、花坛路等城市断头路5条，平桐路泌水河大桥、工业西路泌水河大桥改造工程基本完成。加大污水处理设施投入，新建改建城区供水管网28公里。县文化艺术中心、文化综合体等项目加快推进，泌水河、梁河生态治理工程顺利推进，静脉产业园和城市供热等项目正规划实施。新增绿地面积69.77万平方米。精细管理成效明显。加快推进智慧城市建设，开展“六城联创”活动，加强城区数字化管理，被重新确认为国家卫生县城。农村环境逐步改善。实施乡村振兴战略，推进农村人居环境持续改善。完成农村安全饮水工程33处，效益人口8.63万人。建成通村公路项目37个48.6公里，被确定为省“四好农村路”示范县。农村垃圾收集处理体系基本建立，污水整治有序推进，建成38个乡镇公厕。累计建成达标村216个、示范村120个，分别占行政村总数的61%、34.6%。

（三）脱贫攻坚富有成效。全县累计实现102个贫困村退出、6.97万人脱贫，通过市级脱贫退出初核，有望实现脱贫摘帽。“131”脱贫攻坚责任体系入选全省第一批扶贫干部培训教材，并在全省推广。基础设施不断完善。整合各类财政涉农资金4.58亿元，实施交通扶贫、安全饮水、电网升级改造、环境整治四大工程。投资3000万元占地42亩的县集中托养中心投入使用。全省2019—2020年县级脱贫攻坚项目库建设及统筹整合财政涉农资金培训会在我县召开。产业扶贫全面覆盖。实施集体经济带贫、龙头企业带贫、金融扶贫、网络扶贫、奖补到户增收等8种模式，人均增收在4000元以上。发放金融扶贫小额贷款3.05亿元，带动12493户贫困户。在全省产业扶贫现场观摩评比中获得小组第一名，并作了典型发言，全省网络扶贫现场会在我县召开。政策保障落实到位。贫困对象两免两保和重症慢性病患者门诊就医补助全面落实，报销总比例为95.1%。资助贫困学生1.68万人次，发放资助金1692万元。共为4846名贫困残疾人代办残疾证，为3670名贫困残疾对象补贴30余万元。易地扶贫搬迁后续管理工作在全省作了典型发言，被评为“优秀”档次。完成贫困户危房改造5113户。问题整改实现清零。加强精准识别、精准退出动态管理，突出抓好自评核查，开展县处级领导干部蹲点调研、集中大走访、脱贫攻坚百日会战三大活动，推动问题整改到位。问题整改工作在全省作了唯一发言。在全省2018年脱贫攻坚半年成效核查评估中，我县位居全省第四。

（四）生态建设力度加大。我县2017年度生态文明建设考核位居全市第一，能源消耗总量和强度“双控”目标考核位居全市第二，被评为全省十五个之一、全市唯一一个省级生态县。大气污染防治深入开展。建立“双组长”协调推进机制，成立专项工作组，突出抓好“六控”，持续开展扬尘治理、石材区整治、餐饮油烟污染整治、秸秆禁烧、散煤燃烧等9个专项行动，全年优良天数达261天，比市政府下达的目标净增31天，位居全市第三；PM10平均浓度86微克/立方米，位居全市第一；PM2.5平均浓度52微克/立方米，位居全市第二。全面完成三项市定目标任务。水污染防治持续向好。实施梁河水环境整治、石材区拦截坝等12个重点工程。全面落实河长制，持续开展饮用水水源地保护、非法采砂、畜禽养殖污染等6个专项行动，排查整治河道垃圾点828处、河道排污口337处，关停非法采砂场192处，25家规模化畜禽养殖场全部完成治理任务。饮用水水源地水质达标率100%，地表水出境断面全面达标。土壤污染防治稳步推进。中央环保督察“回头看”、省市督导反馈问题整改到位。生态创建取得突破。完成创森任务15.49万亩，代表全市接受国家林业局创森验收，我县被市委、市政府记集体二等功。累计创建成2个国家级生态乡镇、16个省级生态乡镇、24个省级生态村、93个市级生态村。

（五）改革开放全面深化。在重点改革方面，深化“放管服”改革，持续调整行政职权事项，梳理审批事项856项、服务事项117项。深化商事制度改革，积极推进企业注册登记便利化、“多证合一”、企业登记全程电子化，推行“一网通办”，实现审批服务便民化。38家“僵尸企业”按期完成处置任务。农村产权、盐业管理、国有林场等改革有序推进。在开放招商方面，采取产业链招商、以商招商、驻地招商等方式，先后组织县外招商活动16次，新引进上海洲业国际汽车城、金宝师食品工业基地等亿元以上项目8个，实际利用外资4066万美元，完成目标任务的114.7%，居全市第二；合同利用外资完成4617万美元，完成目标任务的118.7%，居全市第一。在创新驱动方面，新认定高新技术企业9家，17家企业被认定为国家级科技型中小企业，培育市级强优企业12家。认定1家省级、2家市级工程技术研究中心。规模以上高新技术产业增加值增长18%。

（六）民生福祉持续增加。重点民生实事完成预期目标，全县民生支出达到46.2亿元，同比增长21.1%，占财政预算支出的80%以上。社会保障体系逐步完善。城镇新增就业6598人，完成年度任务的143%。为12.98万符合养老金领取条件的群众发放资金1.54亿元。城乡居民基本医疗参保率为90.2%。强化社会救助，发放城乡低保、残疾人补贴、临时救助、医疗救助等资金5.87万人1.37亿元。泌水、花园、古城棚户区改造项目全部开工建设，交付使用保障性住房5530套。社会事业稳步发展。在城区新建学校2所，改扩建学校216所，新增校舍面积4.19万平方米。教育教学成效显著，被评为全市普通高中教学质量先进县、全市初中教学质量先进县、全市职业教育对口升学先进单位，泌阳一高获得“清华大学2018年生源中学”荣誉，泌阳中等职业技术学校被评为国家中等职业教育改革发展示范学校。首届河南泌阳国际马拉松比赛成功举办。义务教育发展基本均衡县创建顺利通过国家验收。加快推进家庭医生签约服务、分级诊疗及县域医共体建设，完成42个标准化村卫生室建设，县妇幼保健院综合楼已完工，新县人民医院综合办公楼全面竣工。文化惠民工程深入实施，80%的行政村建有文化广场。社会大局和谐稳定。“扫黑除恶”专项斗争取得阶段性成效，民族宗教工作持续向好，信访稳定、安全生产和食品安全形势总体向好，公众安全感位居全省42位。

过去一年，政府自身建设取得新进展。切实加强政治建设，全面贯彻党的决策部署，增强“四个意识”，严守政治纪律和政治规矩，坚决落实“两个维护”。自觉接受省委巡视，扎实推进反馈问题整改。切实加强民主法制建设，全面推进依法行政，法治政府建设取得新进展。全面执行县人大及其常委会的决议和决定，自觉接受人大工作监督、法律监督和政协民主监督，认真听取各界人士、无党派人士和人民团体意见，人大代表建议和政协提案办结率、满意率分别达到100%。坚决落实中央八项规定精神，坚决反对“四风”。落实党风廉政建设“一岗双责”，着力解决发生在群众身边的不正之风和腐败问题。审计、司法、统计、气象、人防、史志等工作取得新成绩。

各位代表，过去一年取得的成绩，是市委、市政府正确领导的结果，是县委带领全县人民团结奋斗的结果，是县人大、县政协监督支持的结果。在此，我谨代表县人民政府，向全县人民，向全体人大代表、政协委员，向各民主党派、工商联、无党派人士和各人民团体，向所有关心支持泌阳发展的各界朋友，表示衷心的感谢并致以崇高的敬意！

与此同时，我们也清醒认识到，我县经济社会发展还存在不少困难和问题，政府工作也存在一些差距和不足。主要是：经济总量小，人均水平低，缺少个头大、拉动能力强、产业关联度深的龙头企业，新兴产业支撑不足，产业结构还不够优化；城镇化水平低，以城带乡能力弱，城市基础设施和服务功能不完善，农村人居环境问题依然突出，实现乡村振兴任务繁重；环境污染防治、安全生产任务艰巨，仍需下大功夫；社会民生事业历史欠账较多，教育、医疗、出行等方面还有群众不满意的地方；政府职能转变还不到位，个别部门服务意识不强、服务效率不高等。对此，我们将本着高度负责的态度，直面问题，勇于担当，在今后工作中认真加以解决。

二、2019年重点工作任务

2019年是新中国成立70周年，是全面建成小康社会、实施“十三五”规划承上启下的关键之年。做好今年的政府工作，我们要按照中央、省委、市委、县委经济工作会议的部署和要求，科学判断发展形势，理清发展思路，进一步坚定发展信心，顺势而为，乘势而上，奋力开创高质量发展新局面。

今年政府工作的总体要求是：以习近平新时代中国特色社会主义思想为指导，全面贯彻党的十九大和十九届二中、三中全会精神及中央、省委、市委、县委经济工作会议和市委四届八次全会、县委十二届七次全会精神，持续落实习近平总书记视察指导河南时的重要讲话精神，统筹推进“五位一体”总体布局，协调推进“四个全面”战略布局，坚持稳中求进工作总基调，坚持新发展理念，坚持以供给侧结构性改革为主线，贯彻巩固、增强、提升、畅通的方针，切实加强党对经济工作的领导，着力打好三大攻坚战，着力推进高质量跨越发展，着力加快城乡建设，着力强化基础能力支撑，着力抓好生态建设，着力保障和改善民生，统筹推进稳增长、促改革、调结构、惠民生、防风险、保稳定工作，进一步稳就业、稳金融、稳外贸、稳外资、稳投资、稳预期，增强人民群众获得感、幸福感、安全感，保持经济持续健康发展和社会大局稳定，为全面建成小康社会收官打下决定性基础，以优异成绩庆祝中华人民共和国成立70周年。

今年全县经济社会工作的主要预期目标是：保持“三个同步、三个高于”（即城乡居民收入与生产总值同步增长、生态环境质量与经济质量效益同步改善、社会事业进步与经济发展水平同步提高，生产总值、财政收入、居民收入增速均高于全市平均水平），生产总值增长8%以上，规模以上工业增加值增长8%以上，全社会固定资产投资增长10%以上，社会消费品零售总额增长10%以上，一般公共预算收入增长10%左右，居民消费价格增长控制在3%以内。创新发展取得新成果，加大研究与实验发展经费投入，高新技术产业、战略性新兴产业占规上工业增加值比重达到8%和16%以上。协调发展取得新成效，常住人口城镇化率提高1.5个百分点以上，人均预期寿命持续提高，九年义务教育巩固率不断提高，医疗卫生、文化、体育等社会事业全面进步。绿色发展取得新提升，单位生产总值能耗下降1%以上，主要污染物排放量完成省定、市定目标。开放发展取得新突破，利用省外资金、境外资金和进出口持续增长，营商环境在全省、全市评价排序实现前移。共享发展取得新进步，居民人均可支配收入增长9.5%，城镇调查失业率和城镇登记失业率分别控制在5.5%和4.5%以内。

围绕上述要求和目标，重点抓好以下七个方面工作：

（一）调结构转方式，推进经济平稳增长。全面实施产业强县计划，加快产业结构调整，推动产业转型发展、创新发展。

一要稳定工业经济增长。加强经济运行分析调度和监测预警，强化要素保障，确保工业经济平稳运行。加强产业集聚区建设。围绕产业集聚区晋升三星级，加强基础设施建设，配套建设集中供热、天然气管道、自来水管道、学校等设施，完成固定资产投资51亿元、主营业务收入340亿元、增加值77亿元。抓好产业延链补链。持续推进夏南牛、食用菌、电子电器三大产业集群培育工程，提升产业集群竞争优势。在夏南牛产业上，实施浩宏皮革加工、肉类深加工等项目。在食用菌产业上，持续实施嘉沁食用菌工厂化循环种植与生产加工、中原食用菌交易市场、鲜菇坊食用菌高科技产业园等项目。在电子电器产业上，实施东莞铭普光磁电子产品泌阳生产基地、星际动力新能源电芯生产等项目，确保夏南牛、食用菌、电子电器三大产业产值实现300亿元以上。加快推进科技创新。大力推进工业企业绿色化、智能化、技术化“三大改造”，实现企业转型升级。加大夏南牛产业的技术攻关力度，推进国家级夏南牛工程技术研究中心创建；依托李玉院士工作站，推进食用菌产业技术研发、成果转化。建设一批科技型、创新型企业和平台，培育国家高新技术企业3家、省市级工程技术研究中心3家，加快建立以企业为主体、市场为导向、产学研深度融合的技术创新体系。

二要发展壮大三产经济。坚持生产性和生活性服务业并重，加快现代服务业发展，补齐产业发展短板。加快商务中心区发展。强化基础配套设施建设，完成盘古大道、北二环向东延伸至温泉路、花园路向东延伸至东环一路等建设、绿化、亮化工程，推进农副产品直批电商综合物流博览园、中原食用菌交易市场、上海洲业国际汽车城等项目建设，引进一批文化娱乐、商务服务、专业市场、金融保险等项目，推进商务中心区晋升一星级。加快物流业发展。完善规划布局，加强物流园区基础配套建设，全面完成新邦物流、汇融物流项目建设，积极引进第三方物流等新型业态，整合现有物流企业，推动一批企业入驻物流园区，建立与产业发展相配套的现代物流体系。加快电子商务发展。加大扶持和引导力度，培育一批电商龙头企业，推进电子商务向中小企业、新型农业经营主体应用，加快传统电商和新兴电商融合发展，力争实现电子商务交易100亿元，确保全县乡村物流配送实现全覆盖。加快文化旅游业发展。充分利用我县盘古文化、红色文化、山水文化资源，推进文化旅游业融合发展。制定铜山风景区5A级景区创建规划实施方案，力争铜山风景区创建成国家5A级风景区。加快推进白云山景区4A级创建步伐，完善盘古山景区、五峰山景区基础配套设施，着力提升乡村旅游，力争进入河南省旅游标准化建设试点县。全年接待游客人数450万人次，实现旅游综合收入4.8亿元。促进房地产业发展。认真落实国家、省、市促进房地产业健康发展的政策措施，加快培育房屋租赁市场，解决房地产企业的实际困难，构建房地产业持续稳健发展的长效机制。实施泌水、花园、古城棚户区改造项目，新开工棚户区改造项目764套，逐步构建多主体供给、多渠道保障、租购并举的住房体系。加快金融业发展。加强政府担保、资金周转等融资平台建设，开展金企对接活动4次以上，培育新三板、新四板等企业12家以上，审结非法集资案件4件，进一步优化金融生态环境。

三要促进非公经济发展。着力推动非公企业发展。认真落实《关于促进非公有制经济健康发展的实施意见》，加大龙头骨干企业、高成长性企业扶持力度，培育一批县级强优企业。深入开展非公企业成长促进计划，积极推进“个转企、小升规、规改股、股上市”。广泛开展创业辅导和创业培训，全力扶持小微企业发展，打造一批“专精特新”小微企业，形成大型企业顶天立地、中小微企业铺天盖地、创新企业占领制高地的生动局面。加强企业家队伍建设。落实好《关于营造企业家健康成长环境 弘扬优秀企业家精神 更好发挥企业家作用的实施意见》，加大企业家培训力度，建立优秀企业家表彰制度，切实维护企业家权益，建设一支对党忠诚、诚信守法、善于创造、敢为人先、专业专心、治企有方、兴企有为，推动泌阳高质量跨越发展的企业家队伍。健全服务管理机制。完善县级领导分包企业、联审联批联办等制度，开辟绿色通道，进一步提高落实效率。坚持落实企业服务日制度，各级各部门每月15日要围绕企业需求开展高效服务，协调解决企业生产经营中遇到的困难和问题。加强对产业集聚区和商务中心区企业服务管理，抓好企业固定资产投资、产值等数据统计，做好“四上”企业入库和企业分类管理工作，在项目入驻、经营管理、项目申报等方面提供全程服务。

（二）强功能提品质，推进城市文明提升。以撤县设市为目标，实施百城提质工程，做好“以绿荫城、以水润城、以文化城、以业兴城”四篇文章，加快推进新型城镇化建设进程，着力把泌阳打造成宜居宜业之地。

一要做精规划设计。发挥好规划的引领作用，进一步挖掘我县历史文化，发挥好山水资源优势，注重城市形态、文化传承等规划设计，让城市更有品位和内涵。坚持实施《泌阳县城市总体规划》，编制百城提质工程规划，加快各项市政公用设施、公共服务设施专项规划和控制性详细规划的编制工作，完成县文化艺术中心、县医院周边区域的控制性详细规划和城市设计规划的编制工作。将铜山湖风景区旅游规划纳入县城总体规划组团发展。开展中心城区城市设计工作。做好乡镇总体规划、村庄建设规划的修编和报批工作。严把项目建设修建性详细规划关，确保规划依法依规落实到位。

二要完善城市功能。统筹处理好老城改造和新区建设、地下建设和地上建设的关系，实施27项重点城市提质工程，加快“六大功能区、两大开发带”建设。突出道路建设，完成东环一路、西三环路、盘古山大道向北延伸至北三环等8条道路建设工程，打通范缜路南段、花园路中段、团结路北段、车站路北段等5条断头路。突出水系建设，持续实施泌水河和梁河生态治理项目。突出城市绿化，完成花园路东段、行政路东段等9条道路绿化遗留工程，完成尚东游园、人民公园、移动公司墙外游园绿化遗留及文化路游园、金凤园二期绿化建设工程。突出配套设施完善，全面完成第一污水处理厂和第二污水处理厂提标改造工程，实施第三污水处理厂建设工程；实施产业集聚区和城区规模小区集中供热、生活垃圾处理、静脉产业园建设工程，实施专业市场改造和新建工程，实施人民路与泌水北路交叉口东北角地下停车场建设工程，全面推进新客运站、公交车站建设工程，完成10座以上公厕建设，实施城区临街道路亮化工程，完成盘古山大道特色街改造工程。推进城市功能更加完善，着力提升县城品质。

三要强化融资保障。利用好上级财政金融政策，找准与我县的结合点，采取PPP、债券等方式，完成项目融资22.3亿元。围绕基础设施建设、生态环境治理、污水供水管网建设、医疗教育等领域，实施7个总投资54.99亿元的PPP项目。积极争取土地、棚改专项债券，发行土地债券7.2亿元、棚改债券5亿元。盘活土地经营，新增建设用地1800亩，增加土地收益。加强项目储备策划和推介力度，强化风险防控，为城市建设提供充足的资金支持。

四要深化城市创建。坚持以创促管，把创建作为加强城市管理的有效抓手，进一步完善城市管理机制，全面提升城区规范化、精细化、数字化管理水平。持续开展城市创建活动，培育和践行社会主义核心价值观，突出抓好全国文明县城、全国健康城市等创建，深化文明单位、文明家庭、文明校园创建。着力解决突出问题，围绕垃圾清运、水体改善、扬尘治理等重点，抓好生态环境治“污”；围绕车辆乱停乱放、违章行驶等重点，抓好交通秩序治“堵”；围绕占道经营、乱设摊点、背街小巷等重点，抓好市容卫生治“脏”；围绕办事难、不作为、慢作为等重点，抓好公共服务治“差”，不断提升市民素质和城市文明程度。

（三）抓融合夯基础，推进乡村振兴战略。按照产业兴旺、生态宜居、乡风文明、治理有效、生活富裕的总要求，科学编制实施乡村振兴战略五年规划，推动实现农业强、农村美、农民富。

一要大力发展现代农业。坚持质量兴农、绿色兴农、品牌强农，加快由增产导向转向提质导向，促进农村一二三产业融合发展。加快调整农业结构。按照省委、省政府提出的“四优四化”要求，利用粮食主产县一次性奖励，建设优质小麦示范基地，提高粮食综合生产能力，确保粮食总产达到87万吨以上。继续实施粮改饲工作，调减籽粒玉米种植面积，扩大优质花生、芝麻、饲草、蔬菜、林果、青贮玉米、中药材等经济作物种植面积。扩大无公害、绿色、有机农产品和地理标志“三品一标”农产品的生产规模，统筹推进国家有机产品认证示范区和省级农产品质量安全县创建，创建国家农业综合标准化示范项目8个、省级标准化示范项目2个。放大特色农业优势。发挥夏南牛、泌阳花菇、金铜山烟叶等品牌优势，做优做强特色农业。推进夏南牛扩群增量，实施湖北振泰天龙牧业公司泌阳夏南牛养殖项目，提高规模养殖比重，创建一批国家级、省级肉牛标准化示范场。大力推进食用菌产业转型升级，走规模化、标准化、产业化发展之路，着力发展草腐菌生产，稳定发展代料香菇2.6亿袋。着力提高烟叶生产收购质量，加强专卖市场管理，推动烟叶产业稳步发展。构建农业经营服务体系。采取财政扶持、信贷支持、鼓励引导社会资本进入等措施，加强对农民专业合作社、家庭农场等新型农业经营主体的规范管理，支持其发展壮大。持续推进土地经营权规范有序流转，加强农业社会化服务体系建设，促进农机、农技、农资、农水、植保、防疫、检测等社会化服务组织发展。

二要改善农村人居环境。加强基础设施建设。实施农村公路“通村入组工程”，新建通村公路160公里、桥梁60座，积极推进省道S333改建项目。实施农村安全饮水巩固提升工程，推进羊册镇水厂供水工程，效益人口4.41万人。完成12个电网薄弱行政村整村推进改造工程，保障农民生活用电需求。强化垃圾污水治理。全面实施农村生活垃圾集中收集、清运、处理工程，加快14个乡镇垃圾中转站建设，建立农村生活垃圾收集处理监管机制。开展集中整治行动，重点整治城乡结合部、铜山湖和板桥饮用水源地、主要交通干线及河道等范围，切实解决好垃圾围村等突出问题。抓好污水处理，实施农村生活污水无害化处理试点工程。实施畜禽粪污资源化利用整县推进项目，完成畜禽养殖废弃物无害化处理中心建设，提升畜禽粪污资源化利用水平。持续推进厕所革命。县选取两个乡镇作为示范乡镇，每个乡镇选取一个行政村作为试点，推进6万户农村户用厕所建设和改造。对新建住房，全面配套建设无害化卫生厕所，逐步对已建厕所进行无害化改造。有效防治面源污染。严格控制农药化肥用量，增加有机肥施用量，实现零增长目标。进一步加大农作物病虫综合防治技术推广力度，开展以虫治虫、以菌治菌等生物防治，提高肥料利用率。加强秸秆禁烧和综合利用，做到监管责任、联合执法、宣传引导、经济奖惩四个到位，确保全年实现零燃烧目标。

三要加强乡村有效治理。强化农村基层组织建设，深化平安乡村建设，完善乡村矛盾调解和便民服务机制，健全自治、法治、德治相结合的乡村治理体系。积极推进象河、付庄、盘古撤乡建镇工作，不断提高城镇化率。加强农村规划管理，开展集镇、道路和村庄“三整治”行动，加大对农村破旧庭院、环境卫生整治和村庄绿化美化力度，规范农村建房行为。持续开展农村“两违”动态化巡查，对发现的“两违”现象严格处理。推进移风易俗，培育文明乡风，开展文明户、文明村镇和五美庭院创建活动，确保县级以上文明乡镇达到乡镇总数35%以上、文明村达到行政村总数的30%以上。

（四）重精准强保障，推进脱贫质量提升。在完成全县脱贫摘帽的基础上，开展脱贫攻坚三年行动计划，巩固提升脱贫质量，完成1129人脱贫任务，确保如期全面建成小康社会。

一要坚持产业增收不降。持续实施集体经济带贫、龙头企业带贫、金融扶贫、网络扶贫、奖补到户增收等8种模式，全面落实“2+N”产业扶贫，实现贫困对象稳定持续增收。依托资源禀赋和特色优势，发挥致富能人引领带动作用，兴建一批扶贫就业创业基地，吸纳更多群众就近就业。发展壮大集体经济，实现100个非贫困村集体经济全覆盖。深入推进金融扶贫，创新金融扶贫模式，提高金融扶贫获贷率，健全资金监管和跟踪监督机制。

二要坚持政策保障不变。坚持脱贫不脱政策，确保“两不愁、三保障”落到实处。深入实施“互联网+分级诊疗”健康扶贫工程，完善医疗救助政策，防止因病致贫、因病返贫。积极推进教育扶贫，进一步完善贫困户子女从学前教育到高等教育全方位、无缝式的教育资助政策，不断提升教育质量。强化住房保障，在完成建档立卡贫困户、低保户、分散供养特困人员、贫困残疾人家庭等4类重点对象危房改造基础上，把其他边缘户危房纳入改造范围，实现农村住房全保障；抓好易地扶贫搬迁群众产业发展、就业安置、公共服务等后续扶持，做到“搬得出、稳得住、能致富”。

三要坚持管理力度不减。坚持精准方略，抓好对已脱贫户、拟脱贫户、政策兜底户和强村、弱村的分类管理，实现户籍、教育、健康、就业、社会保险、住房、农村低保、残疾人等信息有效衔接，不断提升精准脱贫水平。围绕今年脱贫目标以及2.6万户6.97万名已脱贫人口返贫风险，制定《关于建立防范返贫机制的实施意见》，完善防范返贫预警机制，确保持续稳定脱贫。强化产业扶贫、基础设施等项目管理，规范项目入库流程，及时开展项目库动态调整，做好财政专项扶贫资金绩效评价。

四要坚持帮扶队伍不散。不断完善“131”脱贫攻坚责任体系，坚持县脱贫攻坚领导小组、各脱贫攻坚专业小组工作机制，加强驻村第一书记和驻村工作队管理，建立村级后备干部队伍和村级党员后备队伍，持续开展夺旗争星活动，不断提升村级组织的战斗力、凝聚力、向心力。深入开展百企帮百村行动，做好“社会扶贫网”推广应用工作，抓好“10.17扶贫日”系列公益活动，构建多层次、宽领域、全方位的结对帮扶格局。

（五）治污染添绿色，推进生态环境改善。认真贯彻习近平总书记生态文明思想，牢固树立“绿水青山就是金山银山”意识，坚持污染防治和生态建设并重，全面完成市定环境污染防治目标任务。

一要深入推进大气污染防治。以打赢蓝天保卫战为重点，突出重点区域、重点企业、重点时段，狠抓以扬尘控制为重点的控尘、控排、控煤、控油、控烧、控黄标车“六控”措施落实，加大道路扬尘治理力度，持续开展工业废气、施工工地扬尘、燃煤散烧管控、柴油货车排气污染、餐饮油烟污染等专项行动，全面启动乡镇环境质量监测站运行、监测和考核，促进全县空气质量不断提升，确保空气质量优良天数超过市定目标，PM2.5和PM10浓度控制在市定目标之内。

二要持续实施水污染防治。坚持全域治理、系统治理、源头治理，认真落实“河长制”“湖长制”，坚决“堵住”排污口、污染源，统筹推进水资源、水生态、水环境、水灾害“四水同治”。加强城区污水管网建设，完成春水、象河等6个乡镇污水处理站和石材区11座超强磁水体净化治理工程建设。打好全域河流清洁攻坚战，持续开展饮用水水源地保护、石材园区、畜禽养殖污染、非法采砂等专项行动，确保省控、市控出境断面水质全面达标。

三要开展土壤污染防治。加强土壤污染治理和农业面源污染防治，抓好土壤污染治理监测，严格危险废物环境监管，推进固体废物处置及综合利用，有序搬迁和依法关停严重污染土壤的企业，努力守护一方净土。

四要加强生态保护修复。以建立良好生态体系为目标，严格执行生态保护红线。开展国土绿化提速行动，实施生态廊道、城郊绿化、乡村绿化、山区生态林建设工程，完成造林任务10.8万亩，争创省级森林城市。编制35条河流生态修复规划，开展湿地生态恢复和综合治理工程建设，完成国家级湿地公园试点验收。加强生态国土建设，落实最严格耕地保护措施，实施矿山绿色修复工程，加强地质灾害防治，做好第三次全国国土调查工作。创建国家级畜牧业绿色发展示范县。启动国家生态文明建设示范区创建工作，积极推进生态乡镇、生态村建设。

（六）抓改革扩开放，推进营商环境优化。更好发挥改革开放牵引作用，进一步激发市场活力和社会创造力。

一要释放全面改革新红利。推进政府机构改革，抓好职能优化、人员转隶和“三定”方案制定，确保3月底前完成改革任务。深化“放管服”改革，规范审批服务行为，压缩审批时限，优化审批流程，推进更多审批服务实现“一次告知、一窗受理、一网同办、一次办妥”，实现企业和群众到政府办理审批服务事项“最多跑一次”。全面完成商务中心区行政服务中心建设，打造全省一流的县级行政服务机构。纵深推进“三十五证合一”改革，全面推行市场准入负面清单制度。持续推进国企改革，完成县缫丝厂、外贸食品厂等4家破产企业处置。完成农村房地一体登记，抓好农村集体经济组织清产核资工作，统筹推进农村“多权同确”，建立权能完整、流转顺畅的农村集体产权制度。

二要实现对外开放新突破。研究制订招商引资专项行动方案，立足我县主导产业发展，紧盯国内外知名企业、行业龙头企业、品牌上市企业，坚持请进来走出去相结合，推动招商引资向选商选资转变，着力招引一批有辐射力、带动力、影响力的重大项目。积极拓展招商渠道，围绕长三角、珠三角和京津等区域，开展定向招商；主动与外来客商“攀亲结缘”，以良好环境推动以商招商；加强与各地商会协会的联系沟通，开展商会招商；鼓励在外工作及务工人员为家乡发展牵线搭桥，开展老乡引商。发挥李玉院士工作站品牌优势，加大主导产业招商力度。完善招商引资政策和工作流程，明确项目包装、信息发布、洽谈签约、规划引领、土地保障、手续办理、开工建设等各环节的责任，建立部门联动、高效审批等招商机制，实现“包装一批、签约一批、开工一批、在建一批、投产一批、储备一批”的良性循环，切实提高项目履约率、资金到位率和项目开工率。力争全年引进亿元以上投资项目5个，引资总额达到130亿元以上，到位资金55亿元以上。

三要打造发展环境新优势。打好优化发展环境“组合拳”，努力打造环境更优、效率更高、活力更足的一流营商环境。打造便利高效的政务环境，强化企业投资审批、环保、金融、人才、信息等公共服务体系建设，为企业生产提供高效、便捷、全套的优质服务。打造公正透明的法治环境，依法严厉打击封门堵路、恶意阻工、强装强卸、强买强卖、吃拿卡要等扰乱企业生产秩序的行为，为企业发展营造安全、宽松的法治环境。打造诚实守信的信用环境，抓好社会信用体系建设，认真落实违法失信“黑名单”制度，使其一处失信、处处受制。打造既亲又清的廉洁环境，构建新型政商关系，做到亲而有界、亲而有度、清而有责、清而有为。

（七）惠民生增福祉，推进社会治理改善。牢固树立以人民为中心的发展思想，坚持办好民生实事，补齐短板，夯实基础，让人民群众享受更多改革发展成果。

一要强化社会保障，提升群众获得感。认真落实就业创业扶持政策，抓好高校毕业生、农村富余劳动力就业和军转干部、退役士兵安置工作，动态消除“零就业家庭”，全年新增城镇就业4600人以上。扎实做好退役军人服务和管理工作。加强就业和社会保障服务体系建设，实现县、乡、村三级就业和社会保障服务平台达标升级。建立统一的社会保险公共服务平台，完善养老、医疗、失业、工伤等保险制度，不断扩大社会保险覆盖面。提高城乡低保、特困人员生活费标准，完成12个乡镇敬老院升级改造工程。继续落实残疾人生活补贴和重度残疾人护理补贴制度，实施残疾儿童康复救助。

二要强化公共服务，提升群众幸福感。持续实施科教兴县战略，在城区新建幼儿园3所、小学2所、初中1所，实施县体育馆综合建设工程，促进中等职业教育发展，实现教育均衡协调发展。深化县域综合医疗改革，持续推进家庭医生签约服务、分级诊疗和县域医共体建设；加快推进新县人民医院建设工程，完成县妇幼保健院建设搬迁任务，完成42个标准化村卫生室建设，继续免费开展妇女宫颈癌和乳腺癌筛查、预防出生缺陷产前筛查和新生儿疾病筛查。深入实施“农村电影放映”“舞台艺术送农民”“农家书屋”等文化惠民工程，全面完成文化艺术中心建设工程，完成乡村两级综合文化服务中心建设任务，加快推进公共文化服务体系建设，争创省级公共文化服务先进县。

三要强化社会治理，提升群众安全感。有效防范政府债务、企业经营和金融风险。完善应急管理体系，加强应急救援队伍建设，提升防灾减灾救灾能力。严格落实安全生产责任制，坚决遏制重特大事故发生。加强市场监督管理，突出抓好食品药品安全监管，创建国家食品安全城市。深入开展“扫黑除恶”专项斗争，加强社会治安立体防控体系建设，有效化解特殊利益群体的信访突出问题和矛盾，确保社会大局和谐稳定。

加强国防教育和国防后备力量建设，支持驻泌解放军、武警部队、预备役部队、民兵和人民防空建设，深入开展双拥共建，推进军民融合发展。支持工会、共青团、妇联等人民团体发挥桥梁纽带作用，注重发挥工商联等社团作用。认真做好民族宗教、外事侨务、档案、史志、气象等工作。

三、着力加强政府自身建设

各位代表，站在新的历史起点，使命召唤我们励志前行，重任要求我们勇于担当。县政府将以机构改革为新契机，按照全面从严治党要求，履职尽责、勇挑重担，履行新使命，展现新作为，不断提高施政能力和水平，切实做到信念过硬、政治过硬、责任过硬、能力过硬、作风过硬。

一要坚持党的领导。坚持以习近平新时代中国特色社会主义思想为指导，深入贯彻党的十九大精神，牢固树立“四个意识”，增强“四个自信”，严守政治纪律和政治规矩，坚决做到“两个维护”。坚决贯彻中央、省、市的各项决策部署，坚决维护县委总揽全局、协调各方的领导，认真落实县政府党组全面从严治党主体责任，确保各项决策部署在泌阳落地生根、开花结果。

二要坚持依法行政。依法接受县人大及其常委会的工作监督和法律监督，坚决落实各项决议、决定并定期报告工作。自觉接受县政协民主监督，认真听取各民主党派、工商联、无党派人士和人民团体的意见。认真办理人大代表建议和政协委员提案，进一步提高办理质量。加强法治政府建设，坚持科学决策、民主决策、依法决策，深化市场监管、生态环境保护、文化市场等领域综合行政执法改革，推动执法责任落实。完善政务信息公开责任清单，推行重大行政决策预公开制度，畅通社会监督渠道。完善公共法律服务体系，深入开展宪法教育，深化“法律六进”活动，进一步增强全社会法治观念。

三要强化责任担当。坚持密切联系群众，落实领导干部分包联系机制，深入基层开展调查研究，着力解决实际问题。完善常态化督查机制，健全限期报告、情况通报、专项督查等制度，严格考核管理，确保重大决策部署落实到位。善于攻坚克难，知难而进、真抓实干，不断提高实现新目标、落实新部署的能力。增强忧患意识，居安思危、勇于担责，有效防范化解各种风险。进一步激励广大干部新时代新担当新作为，保持奋发有为的精神状态，发扬艰苦奋斗的工作作风，做出让人民群众满意的实绩。

四要加强廉政建设。坚决落实中央八项规定精神，驰而不息纠正“四风”问题。严格履行党风廉政建设责任制，坚定不移推进反腐败斗争，保证干部清正、政府清廉、政治清明。健全行政权力约束机制，持续强化对政府投资、工程建设招投标等重点领域、关键环节的监督，从源头上预防腐败，以清廉的政风赢得群众的信任和支持。

各位代表！新时代开启新征程，新征程呼唤新作为。让我们更加紧密团结在以习近平同志为核心的党中央周围，在市委市政府和县委的坚强领导下，不忘初心，牢记使命，砥砺奋进，为实现泌阳高质量跨越发展而努力奋斗，以优异成绩迎接新中国成立70周年！
